# Pregnancy Outcome Using General Anesthesia Versus Spinal Anesthesia for In Vitro Fertilization

**DOI:** 10.5812/aapm.11223

**Published:** 2013-09-01

**Authors:** Azra Azmude, Shahrzad Agha'amou, Fardin Yousefshahi, Katayoun Berjis, Majid Mirmohammad'khani, Farahnaz Sadaat'ahmadi, Kamran Ghods, Ali Dabbagh

**Affiliations:** 1 Department of OBGYN, Tehran University of Medical Sciences, Tehran, Iran; 2 Department of Anesthesiology, Tehran University of Medical Sciences, Tehran, Iran; 3 Research Center for Social Determinants of Health, Department of Community Medicine , Semnan University of Medical Sciences, Semnan, Iran; 4 Department of Cardiac Surgery, Semnan University of Medical Sciences, Semnan, Iran; 5 Anesthesiology Research Center, Shahid Beheshti University of Medicine, Tehran, Iran

**Keywords:** Anesthesia, Spinal, Anesthesia, General, Fertilization

## Abstract

**Background:**

There is a considerable rate of fertility failure and this causes a great burden of untoward effects for patients. Usually a considerable number of these patients undergo anesthesia for their treatment.

**Objectives:**

This study was designed to compare the effects of general and spinal anesthesia on these patients.

**Patients and Methods:**

In a randomized clinical trial, after taking informed written consent from the patients, 200 patients entered the study; 100 in each. During a 2 year period, women aged 20 to 40 years entered the study (one group receiving spinal anesthesia and the other, receiving general anesthesia). Ovum retrieval protocols were the same. Nonparametric and parametric analyses were used for data analysis. P value less than 0.05 was considered significant.

**Results:**

There was no difference between the two groups regarding demographic variables. 15 of 100 patients (15%) in the general anesthesia group and 27 of 100 patients (27%) in the spinal anesthesia group had successful pregnancy after IVF; so, spinal anesthesia increased significantly the chance of IVF success (P value < 0.001; Chi Square).

**Conclusions:**

The results of this study demonstrated that spinal anesthesia increased the chance of fertilization success.

## 1. Background

In 1978 the first successful procedure of in vitro fertilization (IVF) was performed. Today, this procedure has gained an increasing rate with exponential speed, and has been performed in thousands of infertile couples. This clinical method of IVF needs that the physicians, first harvest “mature oocytes from the ovaries of infertile patients” (1). Transvaginal oocyte retrieval guided by ultrasound is an IVF related method performed about 100,000 times in each year in the United States, with similar results in the Europe, and nowadays, it has yielded in more than 3.5 million births all around the world (2). This procedure really takes a very small time interval and physicians usually do it by using general anesthesia, intravenous sedation or local anesthesia (2, 3).

One of the most important issues in obstetric patients is the clinical problems related to infertility (3-5). Among the women in the age range of fertility, there is a considerable rate of fertility failure and this causes a great burden of untoward effects for the patients (6, 7). In vitro fertilization (IVF) is one of the methods used for these cases (8, 9). Usually, patients undergoing IVF tolerate surgical procedures, under general anesthesia or spinal anesthesia (10, 11); so, a considerable number of these patients undergo anesthesia for their treatment (10, 12, 13). Since the administration of intravenous or inhalational anesthetic agents has been claimed to be associated with possibility for pregnancy risk, other routes of anesthesia might help mothers to increase the success rate of pregnancy. This is why administration of anesthesia in pregnant mothers is associated with an increased risk of pregnancy loss (1-3). Though the anesthesia method in the general population does not impose a considerable risk, it might be important for the outcome of IVF (14, 15).

## 2. Objectives

So, this study was designed to compare the effects of general and spinal anesthesia on these patients.

## 3. Patients and Methods

The study was registered in the Iranian Clinical Trial Center "irct.ir" with the registration code of "IRCT138810192804N3". The study plan was approved by IRB ethics committee, Research Deputy, Tehran University of Medical Sciences, Tehran, Iran. The location of the study was a university affiliated hospital in Iran from 2008 to 2010. After taking informed written consent from patients scheduled for elective in vitro fertilization (IVF) in OBGYN ward, 200 were selected and entered the study in a randomized clinical trial method. Sample size calculation and statistical methods: sample size determination was performed after a power analysis in which power = 0.8, α = 0.05 and β = 0.2. After that, the following equation was used to determine the sample size: The sample size (i.e. 200 study patients) was randomly assigned into two groups using simple randomization (the table of random numbers). All continuous data with normal distribution were expressed as mean ± SD and categorical variables were expressed as percentage. The continuous variables were compared using the student t-test. Type of anesthesia (GA vs. spinal), duration of anesthesia (in minutes), and age (in years) were considered as independent variables. Also, other Nonparametric and parametric tests were used for data analysis; including the Kolmogorov-Smirnov, Pearson Chi-square, and Fisher exact tests. All statistical analyses were performed by SPSS software (Version 11.5, SPSS, Inc, Chicago, IL). P value less than 0.05 was considered significant.

The study patients were divided into two groups. So, after using random table of numbers, 100 patients were allocated in each group. For this process, during a two year period, women aged 20 to 40 years were selected and after considering the inclusion and exclusion criteria, entered the study. The inclusion criteria were as follows: elective surgery, informed written consent for performing anesthesia in the form of general or spinal. Exclusion criteria were patient refusal for entering the study, lack of appropriate NPO time, any contraindication for performing spinal anesthesia including patient refusal for spinal anesthesia, spinal deformities, previous spinal surgery, preexisting coagulopathy, platelet disorders, and chronic pain syndromes, history of drug abuse, cardiovascular diseases and uncontrolled hypertension. Also, some factors such as diabetes mellitus, thyroid diseases and smoking may create some effects on success rate; so, they were considered as exclusion criteria of the study.

All the patients had the same therapeutic protocols for ovum retrieval; which was the long protocol. Also, all of them had the same surgical process with the same physicians. For assuring the oneness of the surgeon, all the patients were operated by only one of the surgeons. Also, all the patients underwent the same procedure to ensure the oneness of the surgical procedure. For anesthesia, the first group, received spinal anesthesia for performing In Vitro Fertilization (IVF), and the other group received general anesthesia for this purpose. In the spinal anesthesia group, the patients first were monitored using standard electrocardiography, noninvasive blood pressure, pulse oximetry and heart rate monitoring. Then, 500 mL of isotonic saline was administered, and while a guardian nurse was holding the patient, in the sitting position and under sterile conditions, between the 3rd and 4th lumbar interspaces, a number 25 Quincke needle was introduced and 75 mg of pure 2% lidocaine, without any additive or preservatives was injected into the subarachnoid space. The patients were turned immediately to supine position to gain a T10 (10th thoracic) level of anesthesia. The anesthesia level and the blood pressure were checked continually.

For the general anesthesia (GA) group, each patient was in supine position in operation room. Then, standard monitoring was used including pulse oximetry, pulse rate, noninvasive blood pressure (NIBP) monitoring and 3 lead electrocardiogram monitoring. Also, clinical monitoring of respiratory status was constantly performed. Afterwards, using fentanyl (1 µg/kg) and midazolam (30µg/kg) intravenously as premedication drugs, induction of GA was administered by incremental intravenous doses of thiopental (4 mg/kg) and atracurium (0.5 mg/kg). The trachea was intubated using a low pressure high volume tube (size 7) by direct laryngoscopy. The tracheal tube cuff was filled by air to 15-20 cm-H2O pressure until minimal leak occurred. The anesthesia was maintained by isoflurane (0.8-1 MAC). The residual of muscle relaxant was reversed by atropine (0.03 mg/kg) and neostigmine (0.07 mg/kg) after detection of minimal muscle contractions at the end of the procedure. The patients were extubated in fully awake and stable clinical condition.

Patient evaluation for the results of pregnancy outcome was performed by a different team who were unaware of patient group allocation. The evaluating teams were different from the executive team and evaluated the patients separately at two weeks and six weeks after IVF accordingly. The patients were evaluated regarding the pregnancy outcome using serum β-hCG test two weeks after IVF and sonographic evaluation six weeks after IVF. All the evaluations were performed by the same setting. Pregnancy confirmation was performed after approval of the two tests. The number of fertilized oocytes and embryos transferred in each group and also, the duration in which embryos were outside the controlled environments were kept similar as much as possible.

## 4. Results

There was no difference between the two groups regarding basic characteristics; including age, weight, number of follicles, number of injections of human monocyte gonadotropin (hMG) body mass index (BMI), and duration of anesthesia (Table 1). 

**Table 1.** Baseline Characteristics in the Two Groups

Variables	General anesthesia (N = 100), Mean ± SD	Spinal anesthesia (N = 100), Mean ± SD	P value
**Age, y**	31 ± 5	30.4 ± 5.2	0.382
**Weight, Kg**	67 ± 8.8	67 ± 9.2	0.974
**No of follicles**	8.8 ± 3.2	8.9 ± 3.3	0.933
**No of injections of human monocyte gonadotropin (hMG)**	37.3 ± 10.9	34.7 ± 12.1	0.118
**Body Mass Index (BMI)**	25.9 ± 3.5	25.7 ± 3.6	0.719
**Duration of Anesthesia**	55.5 ± 12.7	58.1 ± 11.2	0.4

The results of pregnancy outcome demonstrated that 15 of 100 patients (15%) in the general anesthesia group and 27 of 100 patients (27%) in the spinal anesthesia group had successful pregnancy after IVF; so, spinal anesthesia increased the chance of IVF success significantly (P value < 0.001; Chi Square).

## 5. Discussion

The results of this study demonstrated that spinal anesthesia can increase the chance of fertilization success. This study suggests that the method of anesthesia could affect the outcome of pregnancy in IVF patient, and this is a new fact which could have important clinical messages accompanied with a number of new questions to be answered in the light of the findings of this study. So, in women undergoing IVF, the anesthesia method would have determining effects, possibly due to the effects of anesthetic drugs and the effects of the anesthesia on the physiologic status of the patients. It has been mentioned that "a combination of propofol, fentanyl, and midazolam is used frequently" in IVF patients with "a relatively low risk for adverse effects on oocyte and embryo quality and pregnancy rates" (1, 2, 16, 17). However, our study demonstrated that the effects of spinal anesthesia on the IVF outcome are even much better. In a study performed on fifty patients scheduled for IVF by Circeo et al, it was demonstrated that “there was no need to use midazolam or larger doses of fentanyl to obtain patient comfort and prevent recall” and this finding implies that patients undergoing general anesthesia or intravenous sedation receive extra doses of anesthetic agents; while there is no need for it and patients undergoing regional anesthesia would not receive such doses to be protected from the anesthetic drug side effects (2). This finding would be in favor of our study. Similar findings have been previously demonstrated by Liu et al (18). In all of these findings, it is explained that “Oocyte retrieval though being a short procedure is preferably performed in ambulatory setting and would necessitate rapid recovery from anesthesia with short recovery and minimal side effects” (2). This study also would be in favor of using regional anesthesia instead of general anesthesia. Besides, it is well determined that general anesthesia is associated with adverse outcomes in IVF for oocyte retrieval (17). There is another study comparing the pregnancy rates among “139 women who received epidural anesthesia, 120women who had paracervical block, and 173 who had general anesthesia with nitrous oxide for oocyte retrieval”; this study demonstrated that “women who had received general anesthesia had significantly lower pregnancy rates compared to the two other groups” (19). This is also in favor of our study to use regional anesthesia instead of general anesthesia.

Conscious sedation and analgesia has been declared as one of several methods used to relieve pain in IVF (20); however, none has been compared with spinal anesthesia and also, none of them has assessed the effect of intravenous anesthetic drugs on the fetus and its survival. This is a very important question and one of the main questions about the anesthetic drugs used for IVF procedures is their effects on the oocyte and the embryo (21, 22). However, when we administer local anesthetics in the subarachnoid space and use spinal anesthesia for these patients, the blood level of the anesthetic drugs is really very much lower (23-25), and this is the most possible explanation for the findings of our study in which the outcome is better for the spinal anesthesia group. This explanation for our findings shows higher chance of IVF success in such patients; this would be in concordance with a number of other similar studies (4, 7, 26).

### 5.1. Study Limitations

If we were able to assess endometrial thickness and it could be considered and defined in each group; it could be a good comparison for assessment between the two groups since endometrial thickness has an important role for success in outcome of pregnancy. However, our study lacks this assessment. Finally, our research demonstrated that in patients undergoing IVF, spinal anesthesia is much more effective than general anesthesia in the success of pregnancy outcome.
